# The global burden of chronic kidney disease attributable to high sodium intake: a comprehensive analysis of trends from 1990 to 2021 and burden prediction to 2040

**DOI:** 10.3389/fneph.2025.1630867

**Published:** 2025-08-18

**Authors:** Yawen Lu, Lei Wang, Jianfeng Ma, Yang Hu, Rumeng Zheng, Liping Liu, Kaili Lin, Kun Zhang, Yongfeng Wang, Sheng Li, Hengping Li

**Affiliations:** ^1^ The First Clinical Medical College of Lanzhou University, Lanzhou, Gansu, China; ^2^ Yanan University Affiliated Hospital, Yanan, Shanxi, China; ^3^ The First People’s Hospital of Lanzhou City, Lanzhou, Gansu, China; ^4^ School of Stomatology Lanzhou University, Lanzhou, Gansu, China; ^5^ Department of Urology, Gansu Provincial People's Hospital, Lanzhou, Gansu, China; ^6^ Graduate School, Ning Xia Medical University, Yinchuan, Ningxia, China; ^7^ Key Laboratory of Molecular Diagnostics and Precision Medicine for Surgical Oncology in Gansu Province, Gansu Provincial Hospital, Lanzhou, Gansu, China; ^8^ National Health Commission (NHC) Key Laboratory of Diagnosis and Therapy of Gastrointestinal Tumor, Gansu Provincial Hospital, Lanzhou, Gansu, China; ^9^ General Surgery Clinical Medical Center, Gansu Provincial Hospital, Lanzhou, Gansu, China

**Keywords:** chronic kidney disease, high sodium intake, global burden, joinpoint regression analysis, age-period-cohort model, future projections

## Abstract

**Background:**

Chronic kidney disease (CKD) is a progressive condition affecting over 10% of the global population, with high sodium intake identified as a critical modifiable risk factor. This study investigated the global burden of CKD due to excessive sodium intake in 204 countries and territories from 1990 to 2021 and made the first future projections to 2040, addressing gaps in longitudinal analysis of sodium-related CKD trends and demographic differences.

**Methods:**

Data from the Global Burden of Disease (GBD) 2021 database were analyzed to quantify CKD-related deaths and disability-adjusted life years (DALYs) linked to high sodium intake. Age-standardized mortality rates (ASMR) and DALY rates (ASDR), alongside the sociodemographic index (SDI), were used to assess regional and demographic variations. Statistical analyses in R included joinpoint regression to identify temporal inflection points and age-period-cohort (APC) modeling to disentangle age, period, and birth cohort effects. Future projections show that from 2021 to 2040, the global ASMR trend is stabilizing and ASDR is on the rise. Moreover, male ASMR and ASDR have been consistently higher than female ASMR. This gender difference is expected to continue for a long time, with men continuing to bear a greater burden of chronic kidney disease than women.

**Results:**

Between 1990 and 2021, global CKD deaths attributed to high sodium intake surged 1.68-fold (26,072 to 69,954), while DALYs increased by 135% (741,197 to 1,705,325). ASMR and ASDR rose markedly in high-income regions (20.73% and 6.77%, respectively), with Latin America and the Caribbean reporting the highest burdens (ASMR: 1.49/100,000; ASDR: 33.21/100,000). Men exhibited consistently higher burdens than women, peaking in the 65–79 age group. Low SDI regions showed declining trends, contrasting with widening inequalities in medium SDI areas.

**Conclusion:**

The global CKD burden attributable to high sodium intake has escalated dramatically over three decades, driven by aging populations, dietary shifts, and regional disparities. Urgent, targeted interventions—such as sodium reduction policies, gender-specific health strategies, and enhanced healthcare access—are critical to curbing this trend, particularly in high-risk demographics and high-income regions.

## 1 Introduction

Chronic kidney disease (CKD) has emerged as a growing global health crisis, currently impacting over 800 million people—more than 10% of the world’s population (1). Alarmingly, these numbers continue to climb year after year. Projections indicate that by 2040, CKD will rank as the fifth leading contributor to reduced life expectancy worldwide (2). While the link between excessive sodium consumption and CKD progression has been established, the full scope of this relationship remains understudied on a global scale. Beyond its devastating health consequences, CKD presents unique clinical challenges, as affected individuals often require particularly complex care (3). The economic toll of managing this condition continues to strain healthcare systems across the globe (4).

Sodium is a crucial mineral that is deeply involved in keeping our bodies running smoothly. Given its vital role, it is no surprise that sodium and health have a Goldilocks-type relationship—you need just the right amount. Too little or too much can lead to health problems (5). Some research indicates that too much salt can throw a wrench into how well your kidneys function (6). When you overload on sodium, your body kicks into high gear to maintain balance (5). The kidneys work overtime to flush out the extra salt and water. However, this constant strain can eventually cause lasting damage, potentially leading to CKD (6). On the flip side, cutting back on sodium can actually protect your kidneys, even if it does not lower your blood pressure (7). Experts suggest aiming for less than 2 g of sodium or 5 g of salt daily, which translates to approximately 90 mmol of sodium excreted in urine (8).

While CKD has been studied extensively, the connection between sodium intake and CKD has not received much attention. There is a real dearth of long-term studies tracking how sodium affects the progression of CKD. Furthermore, this study has not really dug deep into how these risk factors play out differently depending on age and gender. We have seen studies that look at individual risk factors—for example, how high blood pressure (3) affects CKD—but we still need a complete, soup-to-nuts investigation into the global burden of CKD. Considering how little we know about the shifting patterns, the global burden of CKD attributable to high sodium intake highlights the need for better ways to gauge and keep tabs on how the disease burden and health outcomes are changing over time in various groups.

The purpose of this study is to comprehensively investigate the global burden of CKD as determined by the 2021 Global Burden of Disease (GBD 2021) database, as well as the trends in high sodium intake across different categories from 1990 to 2021, including age, sex, social demographic index (SDI) quintiles, GBD subcontinental regions, and countries. We also conducted breakpoint regression analysis using an age-period-cohort model to examine the temporal patterns of the disease over the past three decades. Finally, we made the first future projections for CKD caused by high sodium intake, extending these projections to 2041. These findings provide critical information for formulating policies and initiatives aimed at reducing global high sodium intake, preventing CKD, and protecting high-risk populations.

## 2 Materials and methods

### 2.1 Data source

The research harnessed the GBD 2021 database to paint a picture of the global disease load connected to CKD linked to excessive sodium consumption. The GBD initiative draws on a variety of sources, including census figures, civil records, vital statistics, disease registries, household surveys, health service usage, and air pollution sensors, among other things (9). This all-encompassing strategy allows for a thorough analysis of the CKD burden and its toll on public health. By pinpointing high sodium intake as a risk element, we were able to gather pertinent information on CKD. We also compiled estimates of CKD-related deaths and disability-adjusted life years (DALYs), as well as additional epidemiological data. Every bit of data employed in this venture is accessible to the public.

### 2.2 Basic study variables

We gathered estimates of the CKD burden, including DALYs and mortality figures. DALYs are figured out by simply adding up years lived with disability (YLDs) and years of life lost (YLLs). For the GBD 2021 standard population, we crunched the numbers to get age-standardized rates (average rates per 100,000 people) using the direct standardization method. All age-standardized mortality rates (ASMRs) and age-standardized DALY rates (ASDRs) were adjusted for age. The SDI, a composite measure, is made up of three things: the total fertility rate for the under-25s, the average education level of folks over 15, and the average per capita lagged-distributed income (10). It is on a scale from 0 to 1 and gives you an idea of social and national progress; as the value goes up, so does development. This mountain of data gives us a solid base for digging into how much high sodium intake contributes to CKD around the world.

### 2.3 Study analysis

All statistical analyses were conducted using R software, with a significance level of *p <*0.05 chosen for statistical significance.

#### 2.3.1 Preliminary analysis

To analyze evolving patterns in CKD prevalence over time, we employed least-squares regression modeling along with estimated annual percentage change (EAPC) metrics to quantify shifts in age-standardized incidence rates (ASIRs) during designated observation periods (11). The EAPC serves as a key indicator for tracking ASR trajectories—when both its value and 95% confidence interval (CI) exceed zero, this signals a consistent annual uptick in ASIRs, whereas values below zero reflect a downward trend.

For mapping the worldwide footprint of CKD cases linked to excessive sodium consumption, we leveraged R’s ggplot2 and sf packages (version 4.3.3) to generate detailed cartographic representations, enabling cross-regional comparisons. Drawing from GBD datasets, we visualized geographic disparities in CKD burden attributable to high sodium intake. Our population-level investigation disaggregated male and female prevalence rates by age cohort. Additionally, we examined correlations between SDI and sodium-related CKD impacts. All data processing was performed using R’s dplyr package, with ggplot2 facilitating graphical output.

#### 2.3.2 Joinpoint regression analysis

The joinpoint regression model can identify one or more inflection points in time-series data, segmenting it into intervals. Each interval’s trend is fitted with linear regression to more accurately describe the characteristics of disease changes. The model can be used to investigate the impact of high sodium intake on CKD mortality, DALYs, YLDs, and YLLs. In the joinpoint model, we start with the null hypothesis and fit a simple linear regression model. Changes in CKD-related variables are initially assumed to be linear. We then incrementally increase the number of joinpoints to improve the model fit. Finally, we conduct a permutation test to compare the goodness of fit between the null hypothesis (no joinpoints) and the alternative hypothesis (joinpoints exist). This process helps us select the best-fitting model.

The annual percentage change (APC), the average annual percentage change (AAPC), and their 95% CI are important outcome indicators. In the joinpoint regression model, the APC describes the annual change rate of CKD within each trend segment. The AAPC measures the average annual change rate of an indicator over a specific time span. Their 95% CI can help determine if the study results are clinically significant. If the lower CI limit exceeds the predefined clinical significance threshold, the intervention is deemed clinically valuable; otherwise, it may not be effective. When the *p*-value is below 0.05, the CI aids in further assessing result reliability. A narrow CI that is distant from zero indicates more reliable results, whereas a wider CI near zero suggests greater uncertainty.

#### 2.3.3 The age-period-cohort model

The age-period-cohort (APC) model is a statistical model that can analyze phenomena over time. To conduct APC research, it is necessary to establish a multivariate regression model:


$$Y_{t}=\beta_{0}+\beta_{1}Age_{t}+\beta_{2}Period_{t}+\beta_{3}Cohort_{t}+\in_{t}$$

In a multiple regression model, *Y_t_* represents the dependent variable, while *Age_t_*, *Period_t_* and *Cohort_t_* are independent variables that can predict it. *β*_0_ is the intercept, indicating the expected value of *Y* when all independent variables are zero. $\in_{t}$ is the error term, representing the influence of excluded factors on *Y_t_*.

In this study, age groups and observation periods were divided into 20- and 5-year intervals (12). Mortality and DALYs were examined independently, based on data from 1992 to 2021. We will assess CKD burden trends across age groups, time periods, and birth cohorts in order to uncover the dynamic link between high sodium intake and CKD. This approach not only enhances our understanding of how age, period, and cohort effects influence the relationship between high sodium intake and CKD but also offers a scientific basis for targeted prevention and intervention strategies.

## 3 Results

### 3.1 Global distribution and trends in chronic kidney disease attributable to high sodium intake from 1990 to 2021

As the global obesity rate increased, an estimated 69,954.35 deaths [95% uncertainty interval (95% UI): 7,889.2–198,557.7] attributed to obesity were recorded in 2021, up to 1.68 times more than those reported in 1990 (26,072.12, 95% UI: 4,246.93, 66,433.84). In 2021, global DALY cases due to chronic kidney disease associated with high sodium intake reached 1,705,324.92 (95% UI: 220,083.8, 4,681,197.81), an increase of 135% over 1990. The global ASMR for chronic kidney disease due to high sodium intake was 0.84 per 100,000 in 2021 (95% UI: 0.09, 2.39), and the EAPC was 14.74% from 1990 to 2021. Similarly, the ASDR increased by 6.8% during this period (EAPC: −29.03, 19.18) (**Tables 1**, **2**).

**Table 1:** Death cases and ASMR per 100,00 population of chronic kidney disease attributable to high sodium intake in 1990 and 2021.

Characteristics	1990		2021		1990–2021
	Death cases No. (95% UI)	ASMR per 100,000 No. (95% UI)	Death cases No. (95% UI)	ASMR per 100,000 No. (95% UI)	EAPC (%) in ASMR No. (95% CI)
Global	26,072.12 (4,246.93, 66,433.84)	0.73 (0.11, 1.92)	69,954.35 (7889.2, 198,557.7)	0.84 (0.09, 2.39)	14.74 (−26.06, 28.7)
Central Europe, Eastern Europe, and Central Asia	2,032.16 (457.42, 4,648.64)	0.45 (0.1, 1.04)	3,459.64 (609.06, 8,371.28)	0.53 (0.09, 1.28)	16.69 (−11.59, 32.18)
High-income	5,086.65 (492.96, 15,301.53)	0.42 (0.04, 1.26)	13,795.9 (530.98, 45,520.79)	0.52 (0.02, 1.69)	24.17 (−50.06, 40.01)
Latin America and the Caribbean	2,166.3 (120.11, 6,280.93)	1.15 (0.06, 3.35)	8,975.91 (432.14, 26,627.22)	1.49 (0.07, 4.42)	29.68 (0.19, 47.61)
North Africa and the Middle East	383.49 (0.1, 2,057.91)	0.27 (0, 1.48)	1,304.98 (0.28, 7,337.82)	0.33 (0, 1.88)	24.02 (−56.11, 126.57)
South Asia	2,139.39 (67.32, 7,034.18)	0.43 (0.01, 1.43)	7,738.56 (300.38, 25,010.64)	0.57 (0.02, 1.89)	34.28 (5.85, 136.67)
Southeast Asia, East Asia, and Oceania	12,324.44 (2,826.15, 26,697.38)	1.31 (0.27, 2.94)	30,331.54 (5,754.85, 71,416.33)	1.14 (0.2, 2.73)	−13.36 (−40.46, 3.38)
Sub-Saharan Africa	1939.7 (99.99, 6,362.54)	1.13 (0.06, 3.74)	4,347.83 (116.86, 15,343.49)	1.22 (0.03, 4.32)	8.13 (−50.15, 26.31)
Gender					
Male	14,888.73 (2,718.36, 36,728.29)	0.98 (0.16, 2.50)	41,258.41 (5,491.44, 110,313.72)	1.13 (0.14, 3.05)	0.15 (−0.23, 0.32)
Female	11,183.39 (1,469.36, 30,401.28)	0.56 (0.07, 1.53)	28,695.95 (2,127.65, 87,893.53)	0.61 (0.05, 1.87)	0.10 (−0.40, 0.29)
Sociodemographic index (SDI)					
Low SDI	1,795.34 (94.44, 5,692.36)	1 (0.05, 3.18)	3,831.27 (113.47, 13,219.56)	0.98 (0.03, 3.36)	−1.7 (−51.36, 13.45)
Low–middle SDI	3,760.47 (394.39, 10,649.52)	0.71 (0.07, 2.08)	11,649.55 (785.27, 35,184.71)	0.91 (0.06, 2.8)	27.35 (−25.96, 47.95)
Middle SDI	10,017.04 (1,894.02, 23,472.38)	1.16 (0.2, 2.8)	28,460.22 (3,690.07, 74,413.73)	1.15 (0.14, 3.05)	−1.07 (−37.62, 14.69)
High–middle SDI	5,330.06 (1,064.78, 13,039.03)	0.59 (0.11, 1.5)	12,246.3 (2,072.28, 30,636.39)	0.63 (0.1, 1.59)	5.6 (−19.85, 22.66)
High SDI	5,128.37 (688.25, 14,172.24)	0.46 (0.06, 1.28)	13,688.92 (777.44, 43,392.05)	0.55 (0.03, 1.73)	20.73 (−44.25, 39.98)

**Table 2:** DALY cases and ASDR per 100,00 population of chronic kidney disease attributable to high sodium intake in 1990 and 2021.

Characteristics	1990		2021		1990–2021
	DALY cases No. (95% UI)	ASDR per 100,000 No. (95% UI)	DALY cases No. (95% UI)	ASDR per 100,000 No. (95% UI)	EAPC (%) in ASDR No. (95% CI)
Global	725,260.87 (124,311.77, 1,838,069.14)	18.55 (3.12, 47.36)	1,705,324.92 (220,083.8, 4,681,197.81)	19.81 (2.51, 54.57)	6.8 (−29.03, 19.18)
Central Europe, Eastern Europe, and Central Asia	65,799.96 (13,432.03, 159,239.35)	14.25 (2.91, 34.69)	88,958.78 (14,460.29, 222,349.27)	13.95 (2.28, 35.25)	−2.12 (−27.23, 9.73)
High-income	120,428.85 (14,165.91, 352,239.05)	10.03 (1.18, 29.2)	253,445.48 (10,326.44, 806,963.15)	11.05 (0.47, 34.65)	10.16 (−58.85, 24.95)
Latin America and the Caribbean	54,968.69 (3,168.11, 158,913.12)	25.72 (1.5, 74.5)	205,635.13 (10,343.06, 611,879.08)	33.21 (1.66, 98.54)	29.12 (0.45,46.41)
North Africa and the Middle East	9,828.95 (4.66, 50,759.79)	5.91 (0, 31.37)	32,148.55 (15.14, 176,203.28)	7.13 (0, 39.56)	20.72 (−46.63, 121.33)
South Asia	73,703.12 (2,775.13, 236,530.83)	12.56 (0.4, 41.31)	247,605.03 (12,358.69, 781,215.27)	16.45 (0.74, 52.26)	30.99 (7.41, 119.63)
Southeast Asia, East Asia, and Oceania	353,455.3 (88,499.01, 753,579.54)	32 (7.43, 68.93)	776,640.56 (156,206.38, 1,775,603.94)	27.1 (5.43, 62.61)	−15.31 (−40.08, 0)
Sub-Saharan Africa	47,076.01 (2,332.78, 150,716.3)	23.63 (1.16, 77.34)	100,891.39 (2,526.95, 363,466.49)	23.57 (0.63, 83.44)	−0.25 (−55.86, 17.33)
Gender					
Male	423,298.08 (82,118.91, 1,038,086.63)	23.85 (4.46, 59.34)	1,036,223.17 (155,928.51, 2,709,714.31)	26.08 (3.79, 69.13)	0.09 (−0.24, 0.25)
Female	301,962.79 (44,927.55, 805,882.05)	14.34 (2.08, 38.36)	669,101.75 (60,366.41, 2,007,776.58)	14.46 (1.31, 43.24)	0.01 (−0.43, 0.19)
Sociodemographic index (SDI)					
Low SDI	46201.71 (2264.17,147578.86)	21.82 (1.07,69.87)	95543.58 (2646.35,323953.38)	20.45 (0.58,69.68)	-6.27 (-52.63,8.07)
Low–middle SDI	112167.91 (12388.38,320782.14)	18.5 (1.91,53.75)	321546.95 (23277.87,965256.55)	22.46 (1.55,67.31)	21.43 (-26.95,39.34)
Middle SDI	284540.08 (57141.7,667749.92)	28.14 (5.33,66.75)	719210.46 (105004.97,1847364.2)	26.85 (3.78,68.88)	-4.6 (-39.35,11.02)
High–middle SDI	157146.73 (34246.21,370847.35)	16.07 (3.37,38.64)	306152.43 (58076.91,741209.29)	15.48 (2.94,37.86)	-3.67 (-23.3,10.4)
High SDI	124125.49 (18641.66,340284.61)	11.21 (1.69,30.63)	261115.11 (17114.56,799429.17)	11.97 (0.84,36.25)	6.77 (-50.87,23.56)

Globally, the Deaths and DALYs of CKD attributable to high sodium intake increased in all GBD regions, though declined in Southeast Asia, East Asia, and Oceania between 1990 and 2021. In 2021, in Latin America and the Caribbean, the ASMR (1.49, 95% UI: 0.07–4.42) and ASDR (33.21, 95% UI: 1.66–98.54) were both the highest. The ASMR for men increased from 0.98 (95% UI: 0.16, 2.50) per 100,000 to 1.13 (95% UI: 0.14, 3.05) in 2021, with an EAPC of 0.15% (95% CI: −0.23, 0.32). In contrast, the ASMR for women increased from 0.56 (95% UI: 0.07, 1.53) in 1990 to 0.61 (95% UI: 0.05,1.87) in 2021, with a slight decrease in growth rate (EAPC: 0.10%, 95% CI: −0.40, 0.29). It is noteworthy that the deaths and ASMR numbers remained consistently higher in men than in women, although the increasing trend in male health burden was more pronounced during this 30-year period. Similarly, ASDR followed ASMR and showed similar trends. Strikingly, ASMR and ASDR increased by 20.73% and 6.77% in high-income countries, while they decreased in low SDI regions (ASMR −1.7%, ASDR −6.27%), highlighting different trends across socioeconomic strata (**Tables 1**, **2**).

### 3.2 Global burden analysis

**Figure 1:**
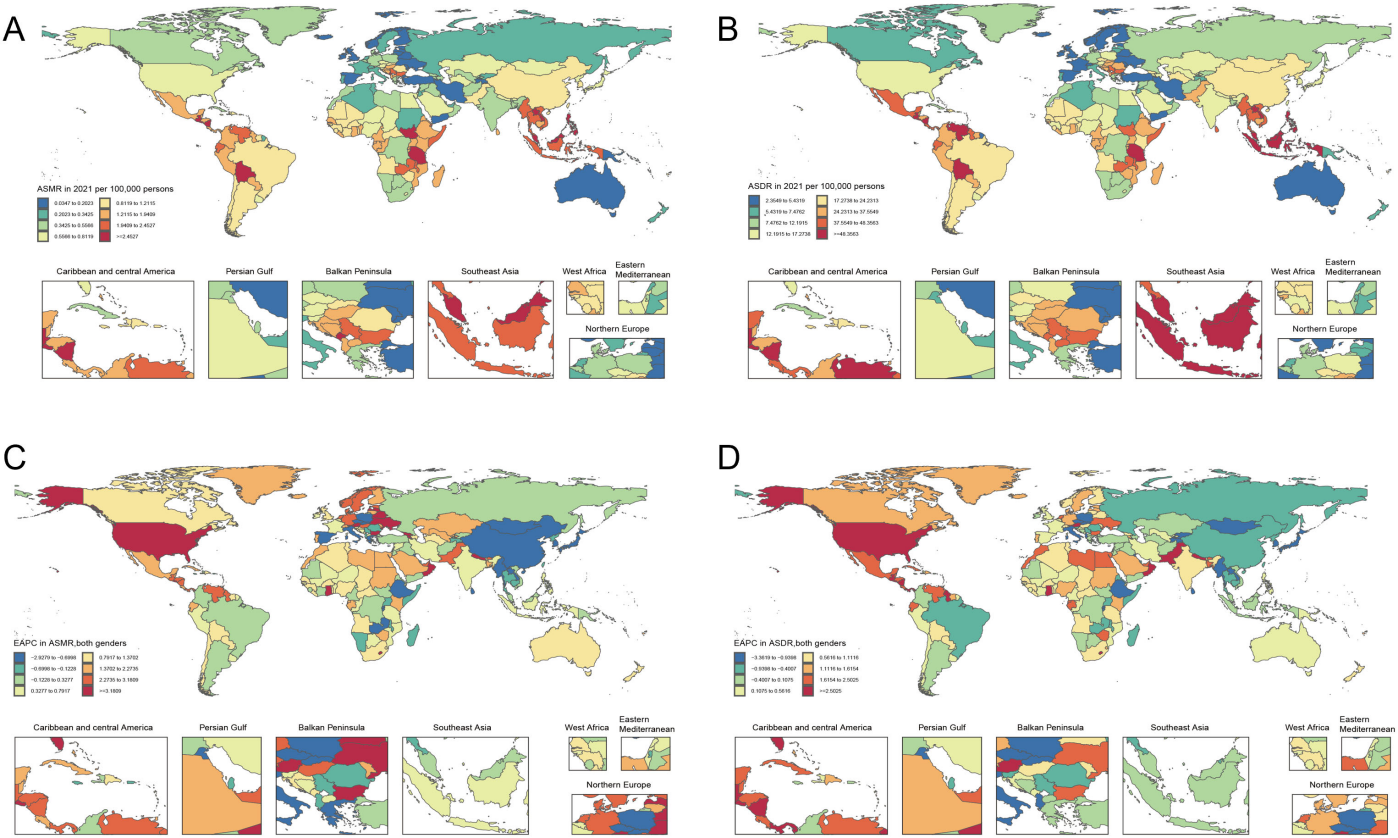
Global distribution of ASR of chronic kidney disease deaths and DALYs attributable to high sodium intake. **(A)** ASMR in 2021. **(B)** ASDR in 2021. **(C)** The EAPC in ASMR, 1990–2021. **(D)** The EAPC in ASDR, 1990–2021.

The global distribution of ASMR and ASDR for CKD due to high sodium intake in 2021 shows significant geographical and national differences. Both ASMR and ASDR burden are mainly concentrated in South Latin America, Central America, Central Europe, Africa, East Asia, and Southeast Asia, with the highest regions (>1.2115 per 100,000 people) including Mexico, Venezuela, Bolivia, Hungary, Central Africa, Central Africa, Tanzania, Lao Guo, Malaysia, Philippines, and other countries (**Figures 1A, B**). The EAPC trends from 1990 to 2021 highlight the dynamic changes. ASMR and ASDR in North America, Northern Europe, Northern Europe, Eastern Europe, North Africa, and Central Asia, including Norway, Sweden, Germany, Netherlands, Belarus, Ukraine, Libya, Egypt, Ghana, Pakistan, Nepal, and other countries (China, Mongolia, Japan, Germany, and Ethiopia) have significantly lower burden (**Figures 1C, D**). It is worth noting that Italy and Russia have been showing a low burden.

### 3.3 Analysis of regional differences and health inequalities

**Figure 2:**
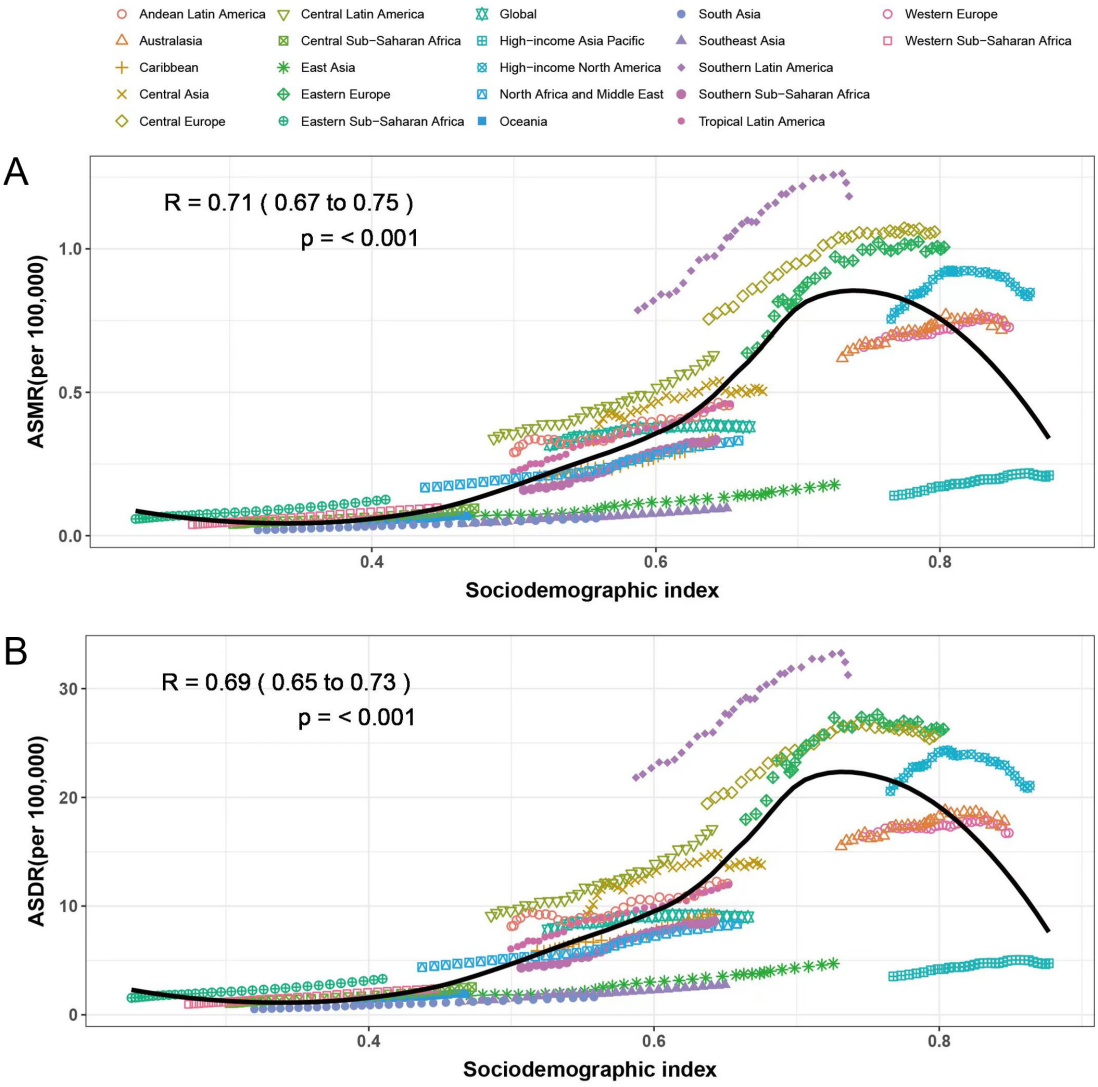
Correlation between ASMR, ASDR, and SDI in chronic kidney disease attributable to high sodium intake for 204 countries and 21 regions, 1990–2021. The above points show estimates for each country and region. The correlation between ASMR **(A)** or ASDR **(B)** and SDI in 21 GBD regions. The association between ASMR **(C)** or ASDR **(D)** and SDI in 204 countries.

The relationship between SDI and ASMR and ASDR of CKD is non-linear both globally and throughout the 21 GBD regions. When SDI was below 0.75, the burden increased significantly with increasing SDI. However, further increases were associated with a decrease in SCI burden. High SDI regions, such as high-income South Latin America, Central Europe, high-income North America, and Australasia, are clustered at the upper end of the ASMR and ASDR ranges. In contrast, regions with low SDI, including eastern Sub-Saharan Africa, western Sub-Saharan Africa, central Sub-Saharan Africa, Oceania, South Asia, and West Asia, exhibited lower disease burden. It is worth noting that despite the high-income SDI region, ASMR and ASDR values were much lower than other high SDI regions (**Figures 2A, B**).

**Figure 3:**
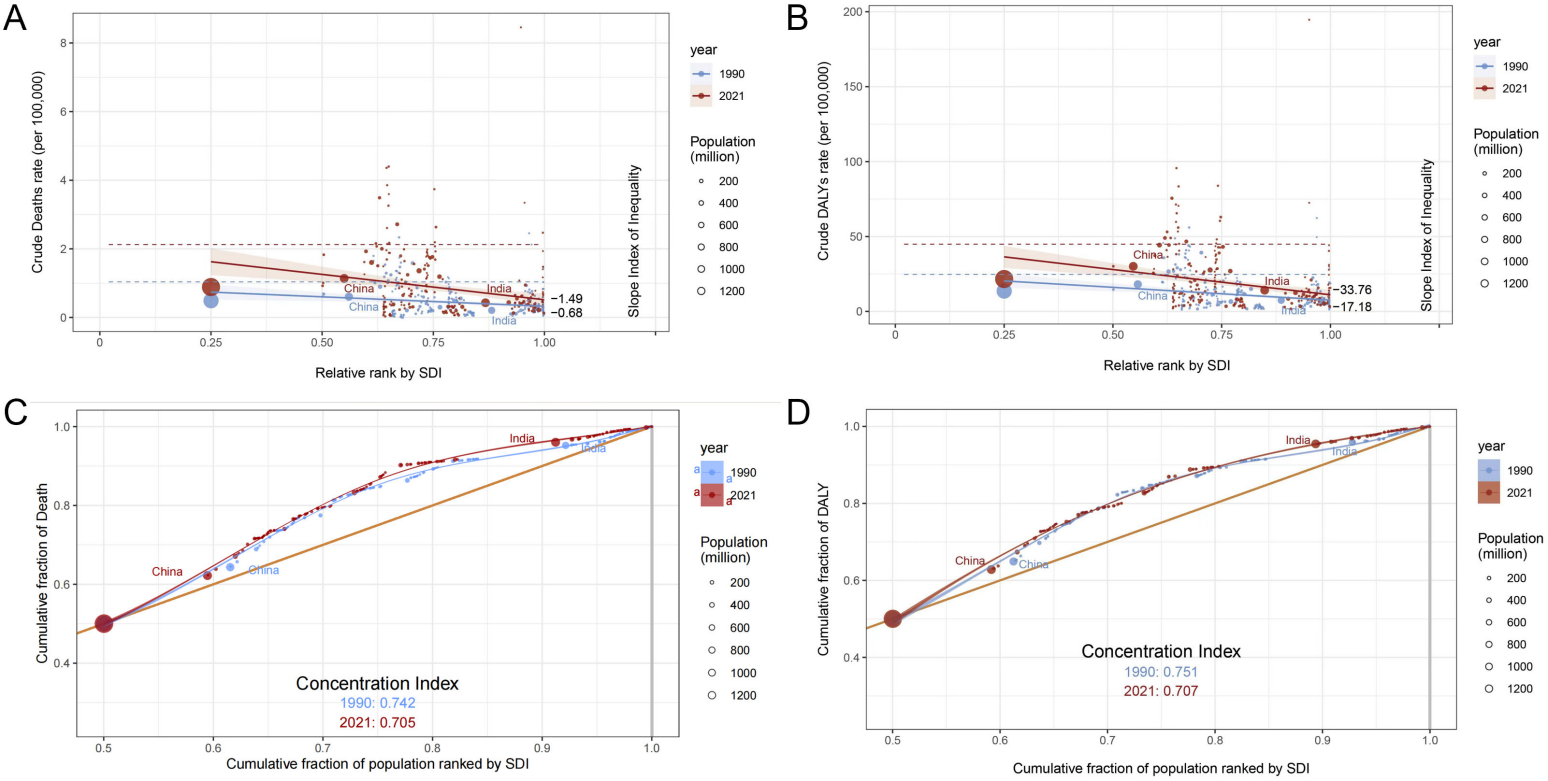
Analysis of death and DALY inequality in chronic kidney disease attributable to high sodium intake in 1990 and 2021. The 1990 and 2021 statistics are displayed in blue and red, respectively. **(A)** Crude death rate per 100,000 by SDI, 1990 and 2021, by relative rank. **(B)** Crude DALY rate per 100,000 by SDI relative rank, 1990 and 2021. **(C)** Cumulative death rate by cumulative population percentage, ordered by SDI in 1990 and 2021. **(D)** Cumulative fraction of DALY relative to cumulative fraction of population, 1990 and 2021, sorted by SDI.

Globally, the burden of CKD due to high salt intake was concentrated in low SDI regions in 1990 and 2021, and inequality worsened among people with different SDI rankings. The health inequality index remained negative from 1990 to 2021 and decreased with rising SDI, indicating a greater burden of CKD in areas with low SDI. The health inequality index for mortality and DALYs decreased from 1990 to 2021 (deaths: −0.68 to −1.49; DALYs: −17.180 to −33.76), indicating worsening inequality status (**Figures 3A, B**). The deaths and DALYs for 1990 and 2021 were consistently large and slightly improved (deaths: 0.742 to 0.705; DALYs: 0.751 to 0.707) (**Figures 3C, D**). China and India have a large population base, and even a reduced change in proportion can have a significant impact on health equality issues. From 1990 to 2021, the proportion of mortality and disability adjusted life years decreased in China, while mortality and disability adjusted life years decreased in India.

### 3.4 Age and sex differences and temporal trends in chronic kidney disease attributable to high sodium intake

**Figure 4:**
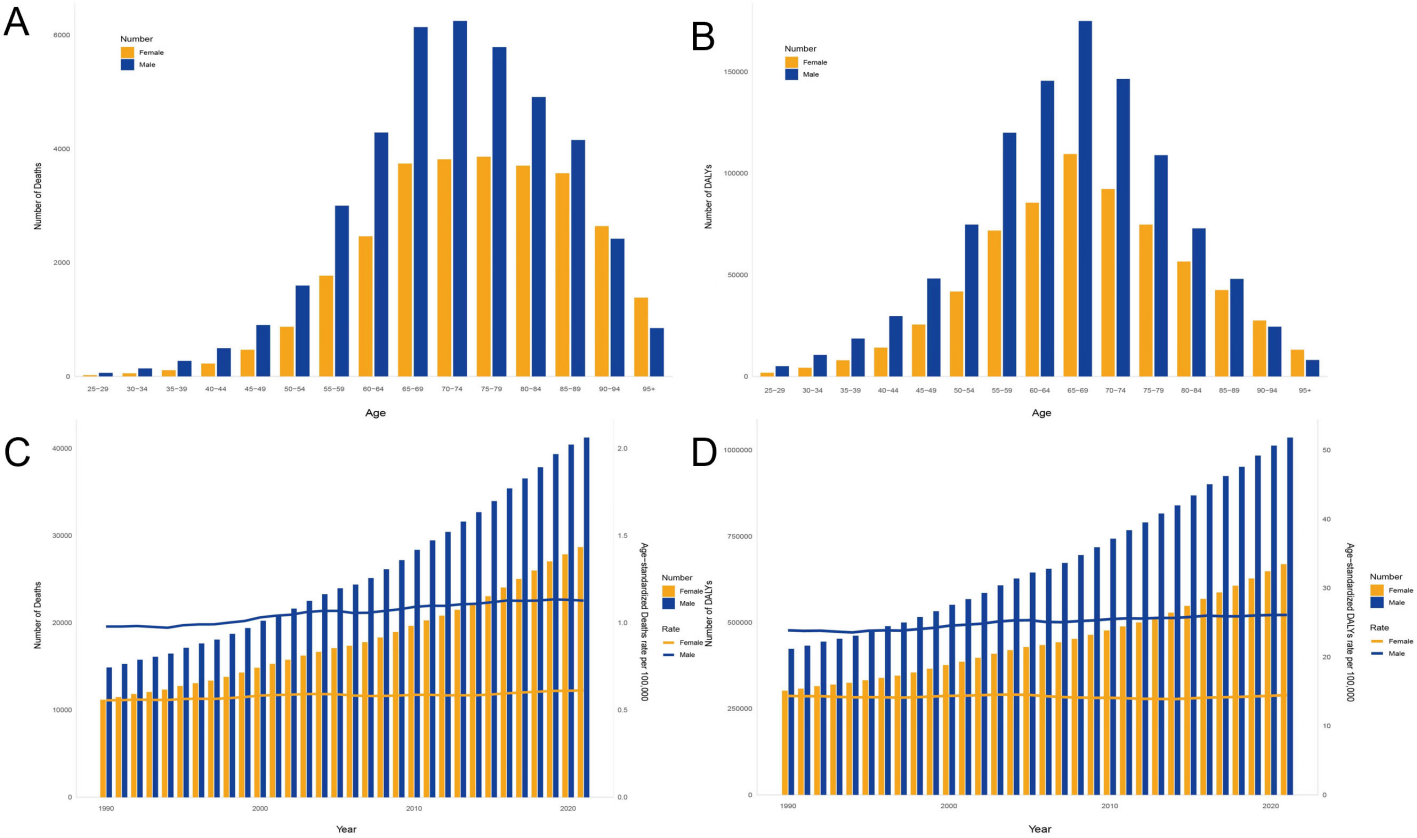
Age–sex characteristics and temporal patterns of the global burden of chronic kidney disease attributable to high sodium intake. **(A)** Deaths in all age groups, 2021. **(B)** DALYs in all age groups, 2021. **(C)** Deaths during various years, from 1990 to 2021. **(D)** DALYs during various years. Disability-adjusted life years, or DALYs, from 1990 to 2021.

The global burden of CKD caused by high sodium intake exhibits different demographic and temporal patterns. The trend of disease burden was higher in men than in women and continued to grow throughout 1990–2021. Death and DALYs were consistently higher in men than in women in almost all age groups, and this difference was more pronounced in the 55–79-year age group (**Figures 4A, B**). In 2021, peak deaths associated with high sodium intake occurred in individuals aged 65–79 years, reflecting the high vulnerability of this age group (**Figure 4A**). However, the loss of disability caused by CKD was more concentrated in the age group of 65–69 years and the lower the age group (**Figure 4B**). From 1990 to 2021, the number of CKD-related deaths and the disability-adjusted life years continued to increase, with steady growth rates, both reaching an unprecedented peak in 2021 (**Figures 4C, D**).

**Figure 5:**
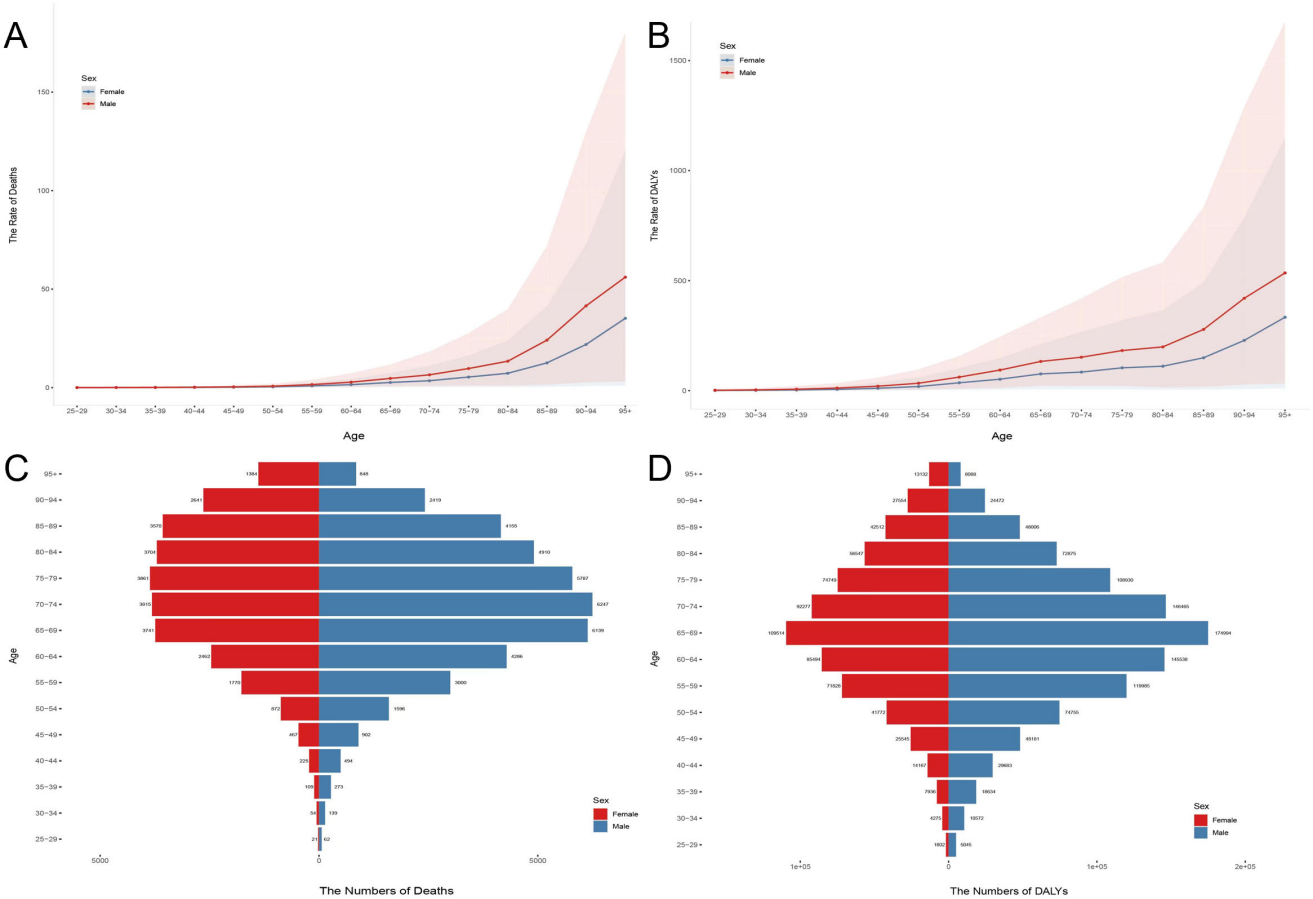
Age and sex differences in chronic kidney disease attributable to high sodium intake in 2021. **(A)** Death rates for women and men in all age groups. **(B)** DALY rates for women and men in all age groups. **(C)** Death cases for women and men in all age groups. **(D)** DALY cases for women and men in all age groups.

The burden of disease increased with age in both men and women, but women were more affected by changes in age. The mortality and disability rates in men and women gradually increased from 50 to 54 years, increased significantly from 80 to 84 years, and peaked in the 95+ years age group. Note a clear gender imbalance in the disease burden of men and women after the age of 80 years (**Figures 5A, B**). The number of deaths and disability loss years increased with age and showed a downward trend in later ages. Male deaths were mainly concentrated in the 65–89-year age group and reached a peak of 3,681 cases in the 75–79-year age group. Female deaths were mainly concentrated in the 65–79 age group and reached a peak of 6,247 cases in the 70–74-year age group (**Figure 5C**). Year of disability loss was more influenced by age than deaths and peaked in the 65–69 age group for both men and women (men: 109,514; women: 174,994) (**Figure 5D**).

### 3.5 Results of the joinpoint regression analysis

**Figure 6:**
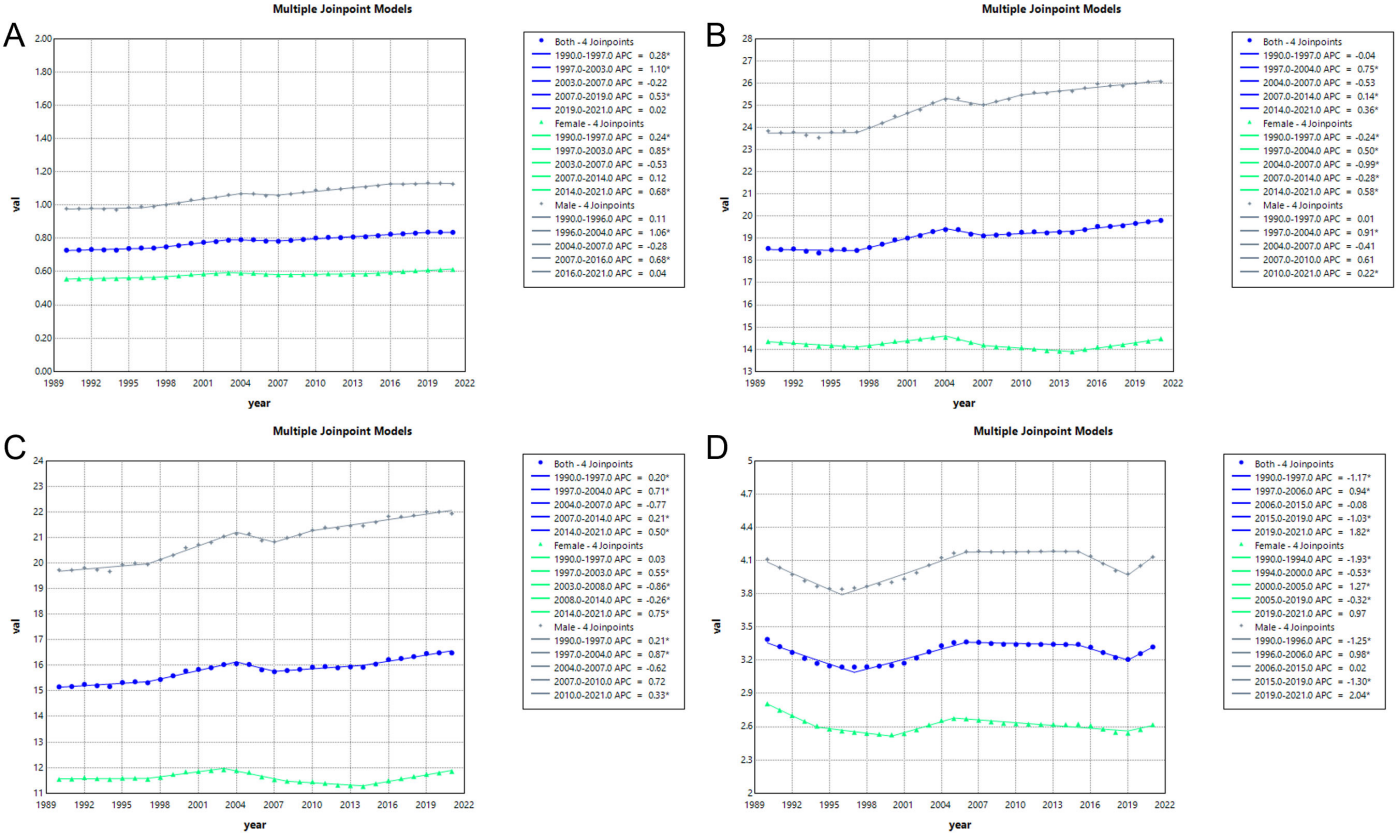
Joinpoint regression analysis of chronic kidney disease attributable to high sodium intake burden temporal trends, 1990–2021. **(A)** ASMR; **(B)** ASDR; **(C)** YLLs; **(D)** YLDs.

Combined point regression analysis showed that ASMR and ASDR were due to high sodium intake in global CKD in 1990–2021. Overall, ASMR and ASDR showed an increasing trend but showed a non-significant downward trend in 2004–2007. The trend of disease burden was roughly similar in men and women, but the burden was significantly greater in men, and the difference between men and women was more pronounced in ASDR. Specifically, the ASMR of CKD increased overall from 1990 to 2021 (AAPC = 0.48%; 95% CI: 0.37% to 0.58%; *p* < 0.001), but the annual growth rate became negative between 2003 and 2007 (APC = −0.18%; 95% CI: −0.81% to 0.45%; *p* > 0.05) (**Figure 6A**, **Supplementary Table 1**). ASDR also showed a global upward trend (AAPC = 0.21%; 95% CI: 0.13% to 0.29%; *p* < 0.001) with the fastest growth from 1997 to 2004 (APC = 0.71%; 95% CI: 0.62% to 0.81%; *p* < 0.001), which turned negative during 2004–2007 (APC = −0.50%; 95% CI: −1.08% to 0.07%; *p* > 0.05) (**Figure 6B**, **Supplementary Table 2**). Similarly, YLLs of CKD continued to rise from 1990 to 2021 (AAPC = 0.29%; 95% CI: 0.19% to 0.40%; *p* < 0.001) but also negative during 2016–2021 (APC = −0.77%; 95% CI: −1.71% to 0.18%; *p* > 0.05) (**Figure 6C**, **Supplementary Table 3**). In contrast, YLDs were more complex and volatile, decreasing against YLLs in 1990–1996 and 2015–2019 and increasing the most in 2019–2021 (APC = 1.82%; 95% CI: 1.38% to 2.27%; *p* < 0.001) (**Figure 6D**, **Supplementary Table 4**).

### 3.6 Results of the age-period-cohort analysis

**Figure 7:**
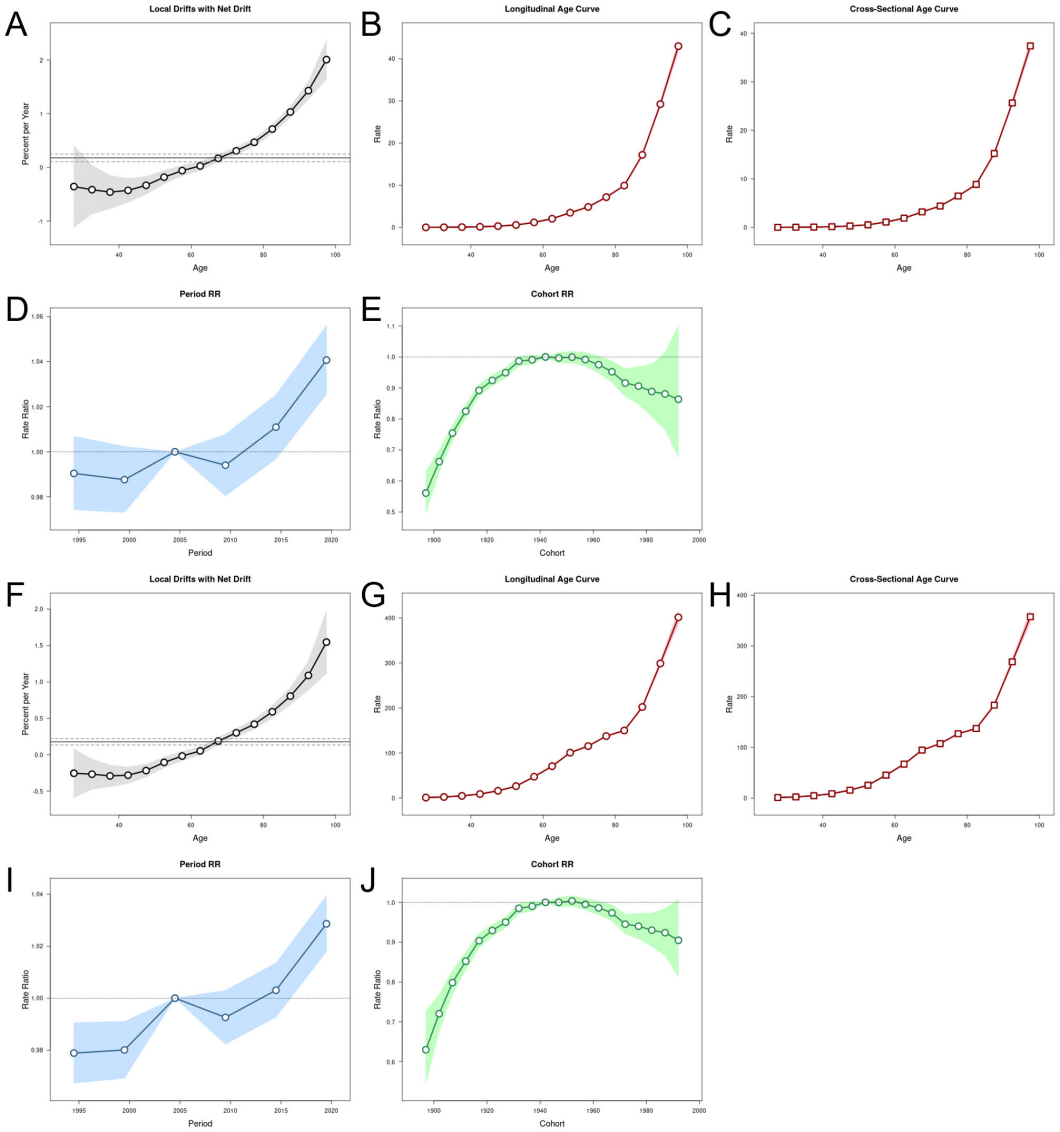
Age-period-cohort analysis results for deaths and DALYs. **(A)** Local drifts with net drifts in deaths; **(B)** longitudinal age curve in deaths; **(C)** cross-sectional age curve in deaths; **(D)** period RR in deaths; **(E)** cohort RR in deaths; **(F)** local drifts with net drifts in DALYs; **(G)** longitudinal age curve in DALYs; **(H)** cross-sectional age curve in DALYs; **(I)** period RR in DALYs; **(J)** cohort RR in DALYs.

For deaths, local drifts showed an annual percentage increase with age, with the most pronounced drift observed in the 80’s population (**Figure 7A**). The longitudinal age curve and cross-sectional age patterns showed that mortality was positively associated with age and rose sharply after the age of 80 years (**Figures 7B, C**). The period effect showed a progressive increase in rates overall, indicating that the effect of high sodium intake on CKD mortality was amplified over time. However, the 2010 ratio was significantly lower than that in 2005 (**Figure 7D**). A cohort analysis found that the risk gradually increased in the birth cohort since 1900 and gradually decreased after reaching its peak in 1950 (**Figure 7E**). DALYs showed a similar trend to deaths in the results of the age-period-cohort Analysis. Notably, the disability loss rate on the longitudinal age curve and the cross-sectional age patterns increase date mortality from age 50 (**Figures 7 F–J**).

### 3.7 Future projections

**Figure 8:**
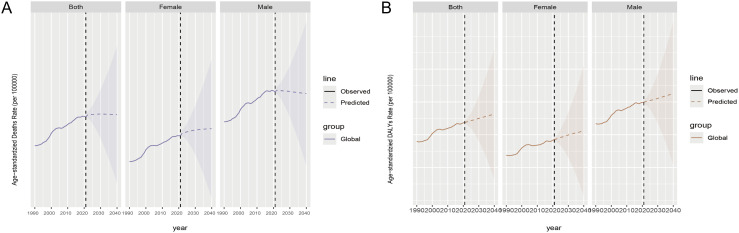
Future prediction by 2040 for chronic kidney disease attributable to high sodium intake, both globally and by gender. **(A)** ASMR; **(B)** ASDR. Observed data are shown as solid lines showing past data. The predicted data are represented as dashed lines showing future trend predictions. The shaded area represents the range of uncertainty in the forecast.

From 2021 to 2040, global ASMR trends are stabilizing, but the differences between men and women are significant. Female ASMR is on the rise, while male ASMR is declining. In terms of ASDR, both globally, among women and men, ASDR is increasing. From 1990 to 2021, male ASMR and DALY rates have consistently been higher than those of females. This gender disparity is expected to persist for a long time, with men continuing to bear a greater burden of CKD compared to women (**Figures 8A, B**).

## 4 Discussion

The global burden of CKD skyrocketed due to excessive sodium consumption between 1990 and 2021. Deaths related to CKD surged 1.68-fold, while DALYs shot up by 135%. Despite these substantial jumps, the increases in adjusted deaths and DALYs were relatively moderate. This pattern aligns with what we observed in the 1990–2019 study (13), and it has only gotten more intense. The reasons for this include shifts in eating habits, population growth, and an aging demographic. In Southeast Asia, East Asia, and Oceania, rising death counts (+146%) contrasted with declining ASMR (-13%). This suggests population growth and aging were primary drivers, rather than increased disease risk per capita. The CKD burden from high sodium levels varies widely across countries and regions, and it is intricately linked to SDI levels. The most severe cases are found in Latin America, Sub-Saharan Africa, and Southeast Asia, where SDI is low. On the other hand, North America and Northern Europe have seen the fastest growth in CKD rates, correlating with higher SDI levels. Over the past 30 years, the burden of disease has been higher in men than in women before the age of 90, and the increasing trend is more obvious. The above findings suggest the need for targeted public health policies and interventions to mitigate the rising burden of CKD associated with high sodium intake factors.

In 2021, the burden of CKD associated with high sodium intake was globally poor, and the variable trends vary across countries. Instead, the low burden areas rose, and the high burden areas showed a low annual growth rate, especially in China, Mongolia, Japan, and other countries, and the burden was significantly reduced. Since 2007, China has really taken the bull by the horns when it comes to lowering sodium consumption. They have done things like fast-tracking changes to nutrition labels on packaged foods to make them more in line with current nutritional science, and they have been working hard to get the word out about healthy eating habits. Just to keep your body running smoothly, you really only need about 200 to 500 mg of sodium each day—anything more than that is often overkill (14). Therefore, the decrease in the average daily sodium intake of Chinese residents from 5.9 g in 2002 to 5.2 g in 2012 (15) may have a positive impact on reducing the disease burden. The burden in Italy has been low, reflecting adherence to the Mediterranean diet pattern and focus on salt intake (16, 17). The low burden in Russia is mainly due to the early diagnosis methods. Researchers at Qiuming National Medical University (TyumSMU), a member of the Russian Ministry of Health, proposed a method using positron emission and computed tomography (PET/CT) to diagnose CKD before kidney function problems (18). This approach evaluates the molecular cell viability of tissues by observing the fixation of labeled glucose molecules in the kidney cells, thereby identifying early CKD beyond any existing diagnostic method. Studies (19) indicate that people typically hang onto their cultural or ethnic heritage through their dietary habits, and this, in turn, shapes their food choices. It is also crucial to realize that where people get their salt from differs drastically from country to country, meaning that different approaches are needed. For instance, in nations where processed foods are the main culprit for high salt intake, the focus should be on creating and enforcing regulations for the food industry. On the other hand, in countries where home cooking is the primary source of salt, we need to put more effort into encouraging individuals to change their habits, as consumer decisions still hold significant sway. By the same token, while policy interventions are always important, we might need to put more stock in strategies that motivate and enable individuals to cut back on salt when they are cooking. These points underscore just how complex and varied attitudes toward sodium reduction can be, and highlight the need for sodium reduction strategies that are customized to a wide range of cultural and sociodemographic factors (20).

SDI presents a non-linear complex relationship with the disease burden of CKD, mainly in low SDI areas, and inequality has worsened among populations with different SDI rankings. Compared with low SDI areas, residents of high SDI areas have easy access to advanced education and better healthcare, as well as coordinated prevention efforts, so the burden is reduced. The Asia-Pacific region is clearly different from other high SDI regions with a low disease burden, indicating the effectiveness of targeted sodium reduction policies. The autonomy of sodium reduction can be improved by improving the overall health awareness of the population or by paying wider attention to nutrition (21). The ASMR and ASDR in the medium SDI regions have exceeded the moderately low SDI and low SDI regions. This region lies between low and high levels of SDI, facing unique challenges related to healthcare infrastructure and disease patterns, such as the transition from infectious diseases to chronic diseases (22). It may also be related to insufficient case reporting and delayed sodium reduction policy in low and medium SDI and low SDI areas. China and India have a large population base, and even a reduced change in proportion can have a significant impact on health equality issues. From 1990 to 2021, mortality and disability-adjusted life year ratios in China declined, while mortality and disability-adjusted life year values in India decreased. It is noteworthy that despite reduced age-standardized rates, China and India remain key factors in the absolute CKD burden due to their large population and continuous high sodium intake. This underscores the dual challenges of addressing population growth and cultural dietary practices.

Men tend to experience a higher disease burden compared to women, partly due to their greater urinary sodium excretion (247 mmol/day versus women’s 218 mmol/day) (23). This physiological difference suggests that men may face increased exposure to high sodium levels, potentially exacerbating health risks. Additionally, men are more prone to engaging in harmful habits like smoking and excessive drinking (24), both of which are well-established contributors to chronic non-communicable diseases. These behavioral patterns further compound the health disparities between genders. Previously, a study showed that smokers preferred salty foods, leading to the occurrence of CKD with high salt intake. A study with 49,558 participants (50.3% women, 49.7% men) (25) showed that men were more likely to die from CKD than women, which is in line with our study results. In addition, the negative impact of exposure to high salt intake was significantly lower in women (26). It is worth noting that, although access to living donor kidneys seems equal between men and women, women reduce the chance of being transplanted by the donor of the deceased (27), so they cannot relax concerns about women’s health issues. This study also showed that the burden of CKD associated with high salt intake was positively associated with age, reflecting the decline in renal function and vascular compliance (28), highlighting the need for targeted intervention and age, especially for the aging population. Early on in life, managing sodium intake can shape one’s taste bud acuity and influence their desire and adaptability to salt as they grow older. Thus, it is vital to prioritize the avoidance of excessive salt consumption among the youth.

Between 1990 and 2021, there has been a noticeable uptick in ASMR and ASDR cases tied to high sodium consumption, which mirrors the growth and graying of the global population. It is given that the incidence of CKD and its risk factors will mirror this aging demographic. We are talking about a scenario where the prevalence of CKD in categories G3–G5 could soar past 10% by 2050, leading to massive health and economic repercussions, especially for low-income nations (29). Heading over to the WHO’s online portal—it is the source where they released their groundbreaking “Global Sodium Reduction” report. It is no secret that we are falling short of our goal to slash sodium intake by 30% by 2025. The WHO is urging member states to act fast and put policies in place to tackle this issue head-on, mitigating the damaging impacts of too much salt. However, it is only nine countries—Brazil, Chile, the Czech Republic, Lithuania, Malaysia, Mexico, Saudi Arabia, Spain, and Uruguay—that have put forth a full range of sodium reduction strategies, making it clear how critical it is to tackle these risk factors. Note that the disease burden showed a clear decreasing trend between 2004 and 2007. Although kidney disease is commonly classified as a non-communicable disease, infection is also an important etiology, directly through kidney involvement (e.g., leptospirosis or HIV infection) or indirectly through infection-associated glomerulonephritis, hemodynamic mechanisms, or systemic inflammatory responses (30, 31). Leptospira seropositivity was a risk factor in Central America in areas with a high burden of CKD in this study (32), potentially contributing to unidentified CKD variants or heightened vulnerability to triggers like heat exposure (33). So, it is reasonable to speculate that the decline in ASMR and ASDR of CKD associated with high salt intake is indirectly affected by the first global public health emergency of the 21st century—the severe acute respiratory syndrome (SARS) virus epidemic in 2003, increasing global awareness of protection and reducing the risk of infection. Although we note that the temporal trend of YLLs is consistent with the DALYs trend, the changes in YLDs are more complex. Post-2019, YLDs soared, highlighting the long-term disability burden and the need for early life intervention to mitigate lifetime kidney damage.

By exposing the differential effects of aging, temporal patterns, and risk in specific birth cohorts, the age-cycle-cohort analysis provides important insights into the temporal dynamics of CKD burden associated with high sodium intake. Mortality and disability loss from CKD associated with high sodium intake increased significantly after age 50 and increased sharply after age 80, emphasizing aging as a key driver of the burden of disease. The period effect showed a progressive increase in rates overall, indicating that the effect of high sodium intake on CKD mortality was amplified over time. Excessive sodium intake will increase the metabolic burden of the kidneys, leading to sodium and water retention, edema, and have a negative effect on kidney function (34) and the rehabilitation of kidney patients. However, the ratio showed a clear downward trend between 2005 and 2007, consistent with the results of the combined point regression analysis in this study and speculated to be due to the indirect effects of the SARS virus epidemic and the global economic crisis. Rising food prices since 2005 (35), and the 2008 financial crisis that caused reduced revenues and higher food costs, have further weakened the ability to prioritize food quality and reduce salt intake (36). The cohort trends showed a decreased risk in the cohort after 1950, reflecting an increased health awareness of CKD due to high sodium intake. A study (37) has shown that the number of salt reduction programs around the world has increased, and more and more countries are choosing structural or regulatory approaches. However, urgent, accelerated, and repeated efforts in other countries, especially in low SDI areas, must require stricter monitoring and evaluation of strategies to achieve salt reduction targets.

Looking ahead to 2040, forecasts regarding CKD linked to high sodium consumption reveal some striking differences between men and women, alongside shifting disease patterns. While the overall death rate appears to be plateauing, the diverging trends—an uptick in mortality for women coupled with a dip for men—suggest underlying variations in biology, lifestyle, and dietary habits. At the same time, the growing prevalence of disability across genders might be somewhat offset by cutting-edge treatments targeting sodium regulation. Drugs like sodium-glucose cotransporter-2 inhibitors (SGLT2i) work by increasing sugar and sodium excretion in urine, which lowers blood volume and pressure—regardless of salt intake (38–41). Meanwhile, next-gen medications such as non-steroidal mineralocorticoid receptor antagonists (ns-MRAs) and aldosterone synthase inhibitors (ASIs) help combat sodium retention caused by aldosterone, possibly protecting the kidneys even when high sodium intake persists (42). But here is the catch: even as death rates level off, age-standardized disability rates keep climbing. This suggests that the long-term kidney damage from excessive salt consumption could eventually outweigh the benefits of these therapies, especially in vulnerable groups. Loading up on salt really throws a wrench into kidney function by worsening protein in the urine and overworking the kidneys’ filters (43). This pushes kidney disease forward, even if blood pressure is under control. It just goes to show how bad dietary habits can lead to long-term health problems, even if they do not seem immediately life-threatening. Popping RAAS inhibitors might buy some time before end-stage renal disease kicks in, but it could also mean a longer period of being disabled (44). Plus, salt-related vascular problems, like an enlarged heart (45), add insult to injury when it comes to CKD. If we are serious about tackling the growing problem of CKD, we need to get serious about public health initiatives aimed at cutting down on sodium consumption. In China’s countryside, a primary focus should be placed on trimming the salt shaker in home-cooked meals. Across the pond in Japan, the UK, and the States, cutting down on the sodium in packaged goods is a no-brainer (46). Moreover, let us not forget the gender gap—specific health services need to be beefed up, from diseases screenings for the fairer sex to spreading the health gospel among women and upping men’s health game. Men and women face different health hazards, so it is crucial to have shrewd policies and better medical service frameworks to lessen the diseases’ toll on our health and lift the health bar for everyone on the planet. Moreover, the aftermath of diseases, like disabilities, needs a close look. We should fine-tune the full spectrum of disease prevention, treatment, and rehab care. Internationally, we need to beef up community rehab centers, train rehab professionals to a higher standard, guarantee that patients get quick and efficient rehab after their recovery, and slash the number of years cut short by disabilities.

While this study provides a comprehensive analysis of the global burden of CKD caused by high sodium intake and makes future projections for the first time, some limitations should be acknowledged. The study relies on data from the GBD 2021 database. Variations in data quality and reporting standards across countries may affect the accuracy of our estimates, particularly in low- and middle-income countries where data collection systems may be less robust. The study assumes a direct causal relationship between high sodium intake and CKD. However, sodium intake is often estimated based on dietary surveys, which may not fully capture individual consumption patterns. Additionally, other dietary and lifestyle factors (e.g., potassium intake, physical activity) that could influence CKD risk were not accounted for in this analysis. While the study identifies trends over a 30-year period, it does not account for potential confounding factors such as changes in healthcare access, advancements in medical treatments, or shifts in dietary patterns over time. These factors could influence the observed trends in CKD burden. The findings are based on global and regional aggregates, which may not fully reflect the heterogeneity within individual countries or subpopulations. Localized studies are needed to tailor interventions to specific contexts.

## 5 Conclusion

The study highlights a significant global rise in CKD burden due to high sodium intake, with marked disparities across regions and demographics. Targeted interventions, such as sodium reduction policies and public health campaigns, are essential to mitigate this growing health challenge, particularly in high-risk populations. Addressing dietary habits and improving healthcare access can help reduce the future impact of CKD.

## Data Availability

The datasets presented in this study can be found in online repositories. The names of the repository/repositories and accession number(s) can be found in the article/**Supplementary Material**.
